# Shifting of Clinical Researches From Statistical Significance to Clinical Relevance

**DOI:** 10.1111/jocd.70456

**Published:** 2025-09-15

**Authors:** Yashdeep Singh Pathania

**Affiliations:** ^1^ Department of Dermatology, Venereology and Leprology All India Institute of Medical Sciences, Rajkot Gujarat India

**Keywords:** anchor‐based method, clinical relevance, MCID

Dear Editor,

Utmost of the researchers of any field including dermatology have reckoned upon the “*p* value” which is considered a statistical corner where value below 0.05 is targeted to draw the results of treatment effectiveness in clinical trials. Statistically significant difference is a numerical value which depicts that the difference is not by chance and is often augmented in researches with larger sample size. The significant statistical value does not inevitably mean that the study or research would have a clinical impact on the case. The clinical difference is a factual change for the case who can perceive as applicable. The term clinical connection is truly delicate to define and quantify.

As clinicians, whether conducting a study or critically analyzing, the clinical difference should have an upper hand in comparison with absolute statistical data. There should be a combination of clinical inference and statistical analysis to illustrate the minimal clinically important difference (MCID), with a part to fill the gap between statistical and clinical significance. The term MCID is the smallest difference of score in the sphere of interest which patients or cases perceive as salutary in the absence of worrisome side effects or expensive costs and which would elucidate a change in patient management [1]. It is a threshold value for the lowest change in the patient‐reported outcome measure (PROM) which is worthwhile to the case.

The MCID evaluates if a patient really feels better or worse after the treatment. Researchers or clinicians have been trying to decide MCID precisely. There are three styles of inferring MCID: expert agreement by Delphi system, the anchor‐based, and distribution‐based styles. The Delphi method is a structured process of gathering expert opinions through rounds of questionnaires and reaching a consensus, which constitutes a meaningful change in the measure under consideration. The anchor‐based method is more oriented to clinical status, and the distribution‐based method is more mathematical, related to statistics. For example, MCID may be evaluated on the basis of standard deviation of the outcome measure or standard error of measurement [2]. The anchor‐based has been used more constantly, where the smallest change in scores would correspond to a fated threshold of change in the external anchor, for example, patient‐reported outcome measures similar to quality of life (QOL) scores. For example, a dermatological disease having a validated score is chosen for analysis of change in disease severity. A responder threshold score is selected as an anchor, such as quality of life score, Likert scoring scale, or visual analogue score, owing to their relevance in other previous studies. Therefore, any change above the threshold score indicates a clinically significant change in disease severity. MCID, apart from being useful in PROM, may also be of help in calculating the power of the study and estimating sample size in clinical studies or trials.

Albeit, MCID systems results which are meaningful to cases, but there are fallacies too where MCID outgrowth styles may induce inconsistent different values which may warrant standardization. Still, understanding the conception of MCID is vital to assay and interpret results of clinical interventions and interpret the results which may not be statistically significant but have a significant clinical connection.

## Author Contributions

**Yashdeep Singh Pathania:** preparing and finalizing the manuscript.

## Ethics Statement

There are no patients involved and adherence to relevant ethical guidelines is confirmed.

## Consent

The author has nothing to report.

## Conflicts of Interest

The author declares no conflicts of interest.

## Data Availability

The data that support the findings of this study are available from the corresponding author upon reasonable request.
